# “Near-Miss” Obstetric Events and Maternal Deaths in a Rural Tertiary Care Center in North India

**DOI:** 10.7759/cureus.11828

**Published:** 2020-12-01

**Authors:** Vandana Verma, Vaibhav Kanti, Soniya Vishwakarma, Umesh K Gupta, Pragya Shree

**Affiliations:** 1 Obstetrics and Gynecology, Uttar Pradesh University of Medical Sciences, Etawah, IND; 2 Pediatric Surgery, Uttar Pradesh University of Medical Sciences, Etawah, IND; 3 Obstetrics and Gynecology, Kanti Devi (KD) Medical College, Hospital and Research Center, Mathura, IND

**Keywords:** severe acute maternal morbidity, haemorrhage in pregnancy, mortality index, quality of health care

## Abstract

Introduction

Maternal near-miss and maternal mortality cases have common characters, especially in terms of risk factors. Both of them are indicators of the quality of health care services provided to pregnant women. Our center is a tertiary care center in a rural area of western Uttar Pradesh (U.P.) so we get a large number of referred cases from most of the rural areas of western U.P. and the adjoining areas of other states too, which sometimes end up in mortality. Thus this study was planned to find out the incidence of maternal near-miss events and compare the nature of near-miss events with maternal mortality.

Goal and objectives

The main objectives of the study were to determine the frequency of maternal near-miss events, observe the trend of near-miss events, and compare the nature of near-miss events with maternal mortality.

Materials and methods

It was a retrospective study conducted in the department of obstetrics and gynecology at Uttar Pradesh University of Medical Sciences (UPUMS), Saifai, Etawah, from July 2018 - June 2019, over a period of one year. Potentially life-threatening conditions and maternal mortalities were noted from the records of the hospital after taking ethical clearance from the institute. Near-miss cases were noted based on the Health and Family Welfare Government of India guidelines 2014. Data were collected and statistical analysis was performed using the Statistical Package of the Social Sciences (SPSS) version 21 (IBM Corp., Armonk, NY).

Results

The maternal near-miss incidence ratio was 16.6/1000 live births, the maternal near-miss to mortality ratio was 1.9:1, and the mortality index was 0.34%. Hypertensive disorders of pregnancy were the most common causes of near-miss events (45.8%) followed by hemorrhage (23.6%) in this study.

Conclusions

Hypertensive disorders in pregnancy and hemorrhage were the two leading causes of near-miss events and mortality followed by sepsis. As the near-miss analysis indicates, the quality of health care and causes are almost similar to maternal mortality, so its registry should be done along with maternal mortality.

## Introduction

Maternal near-miss (MNM) is a condition in which a woman nearly dies from complications of pregnancy or childbirth within 42 days of termination of the pregnancy regardless of location or duration but survives either due to the good care she receives or due to chance [1]. Maternal mortality is one of the standard indicators to assess the quality of services provided by a health care system [2]. But the quality of services provided by the health care system to pregnant women is not only indicated by maternal mortality alone but also by a maternal near miss. The concept of “near-miss” obstetrical events or severe acute maternal morbidity (SAMM) and the criteria to evaluate these cases was given by the World Health Organization (WHO) in 2009 [2]. Worldwide, there is a continuous fall in the maternal mortality ratio (MMR), and it dropped by 38% from 342 in the year 2000 to 211 in the year 2017 per 100000 live births [3]. The MMR in India is also declining; it has declined from 130 during 2014-2016 to 122 during 2015-2017, whereas in Uttar Pradesh, the MMR was 216/100000 live births [4] in 2015-2017. MMR in India is far from the target to be achieved in sustainable development goals (SDG) for which countries have united. In SDG 3, we have to reduce the maternal mortality ratio to up to 70 per 100000 live births [5]. Many states in India have achieved it but most of them are far from it [5]. So to achieve national and international targets, we have to empower our health resources. Extensive studies on “near-miss” obstetrical events have been done in recent years along with maternal mortality. Women who died were part of these near-miss cases at one point in time but due to the delay in seeking health care or other reasons, mortality occurred so they have many common characteristics particularly on the risk factors. To improve our health care system in terms of availability of investigations, equipment, and manpower, a registry of near-miss cases can give valuable information regarding shortcomings in the healthcare facilities of pregnant women, which will help us identify the need for improvement in the referral facility and the need for health awareness programs. In 2014, the Government of India Ministry of Health and Family Welfare also gave guidelines based on WHO criteria to identify MNM cases and their registry [6]. By the effective implementation of the guidelines and the near-miss concept of WHO, we can analyze the high-risk group, plan and execute the required intervention for obstetrical emergencies, and make awareness programs for better outcomes.

Our center is a tertiary care center in a rural area of western Uttar Pradesh so we get a large number of referred cases from most of the rural areas of western U.P. and the adjoining areas of other states too, which sometimes land up in mortality so we planned this study to find out the frequency of maternal near-miss events and compare the nature of near-miss events with maternal mortality.

## Materials and methods

It was a retrospective study conducted in the department of obstetrics and gynecology, UPUMS, Saifai, Etawah, from July 2018 - June 2019 over a period of one year. Potentially life-threatening conditions related to pregnancy, delivery, and maternal mortalities were noted from the records of the hospital after taking ethical clearance from the institute. Near-miss events were noted based on the Health and Family Welfare Government of India Guideline 2014. Data were collected. Statistical analysis was done by using the Statistical Package of the Social Sciences (SPSS) version 21 (IBM Corp., Armonk, NY). The following indices were calculated:

Maternal near-miss incidence ratio-total no of near-miss cases/total no. of live births X 1000

Maternal near-miss/maternal mortality ratio

Mortality index = no. of maternal deaths /no of maternal deaths + no of near-miss cases

Maternal mortality rate

The maternal near-miss to maternal mortality ratio and the mortality index tell about the quality of care given at a particular institute. The higher the maternal near-miss to maternal mortality ratio, the better is the care at the given institute. A lower mortality index signifies better care at the institute.

## Results

In the study period of one year, the total number of deliveries was 8793 and the total live births were 8638. The total no of near-miss cases or SAMM was 144, and 77 maternal mortalities occurred during the study period of one year. The maternal near-miss incidence ratio was 16.6/1000 live births, maternal near-miss to mortality ratio was 1.9:1, and the mortality index was 0.34%.

Table 1 shows the comparison of the demographic profiles of the cases. Most of the women in this study belonged to the age group of 20 to 35 years in both maternal mortality as well as maternal near-miss cases (84.41% and 67.3%). A statistically significant difference (p-.02) was present between age groups between the near-miss and maternal mortality cases. Multiparous women were more in number in both maternal near-miss cases and maternal mortality (44.1% and 58.4%). Maternal mortality was significantly high (p-value 0.01) in unbooked women as compared to near-miss cases than in booked cases (79.2% vs 63.1%). Even the booked cases had no proper antenatal records and lacked in investigations. Ninety-two percent (92.5%) of cases in maternal mortality and 88.7% of near-miss cases were referred from different private and government facilities of Etawah and nearby districts. The number of near-miss cases and maternal mortality both were more in the third trimester of pregnancy (48.5 vs 12.8), and this difference was statistically significant (p-value .01).

**Table 1:** Comparison of demographic profile in maternal near-miss and maternal mortality cases

Demographic characters		Maternal near-miss cases (n=144)		Maternal mortality (n=77)		P-value/degree of freedom/χ^2 value^
		Number	Percentage	Number	Percentage	
Age	<20 yrs	21	14.5	5	6.4	P=0.02 D.F.=2 χ^2 = ^7.48
	20-35 yrs	97	67.3	65	84.41	
	>35 yrs	26	18.0	7	9.0	
Parity	Primimipara	65	45.1	32	41.0	P=0.60 D.F.=1 χ^2 ^=0.26
	Multipara	79	44.1	45	58.4	
Booking status	Booked	53	36.8	16	20.7	P=0.01 D.F.=1 χ^2^=6.00
	Unbooked	91	63.1	61	79.2	
Gestational age	<12 wks	12	8.3	1	1.3	P=0.01 D.F.=4 χ^2 = 11.7 ^
	12-28 wks	16	11.1	4	5.1	
	28 wks-32 wks	21	14.5	6	7.7	
	>32 wks	49	34.0	29	37.6	
	Postnatal	46	32.0	37	48.1	

As shown in Table 2, hypertensive disorders of pregnancy were the most common near-miss event (45.8%) followed by hemorrhage (23.6%) in this study. Hemorrhage was the most common cause of maternal mortality (32.4%) followed by a hypertensive disorder of pregnancy, sepsis, and severe anemia (31.16, 22.0%, and 5.2%), respectively. But if we collectively see rupture ectopic pregnancy and rupture uterus along with other causes of hemorrhage, the most common cause of near-miss, as well as maternal mortality, were both a hemorrhage. Most of the cases of post-partum hemorrhage leading to maternal mortality were referred and reached to us in late stages.

**Table 2:** Comparison of causes of near-miss events and maternal mortality

Causes		Near-miss event (Number=144)	Percentage	Maternal mortality (number=77)	Percentage
Hypertensive disorder of pregnancy (N=66)	Antepartum eclampsia	33	22.9	12	15.5
	Postpartum eclampsia	21	14.5	10	12.9
	Severe preeclampsia	12	8.3	02	2.5
Hemorrhage (N=34)	Antepartum hemorrhage (APH)	12	8.3	8	10.3
	(Postpartum hemorrhage (PPH)	22	15.2	17	22
Sepsis		6	4.1	17	22
Ruptured ectopic pregnancy		9	6.25	1	1.2
Rupture uterus		13	9.02	2	2.4
Severe anemia		11	7.6	4	5.2
Jaundice		4	2.7	2	2.4
Heart disease		1	0.7	0	0
Pulmonary embolism		0	0	2	2.4

Out of all near-miss cases, 92 (63.8%) patients required ventilatory support, 35 (24.3%) patients required vasopressors, 12 (8.3%) patients required hysterectomy for different indications like rupture uterus, placenta accrete, and intractable post-partum hemorrhage not responding to medical and other surgical management, 58 (40.2%) patients needed > 5 units of blood transfusion, and two (1.3%) patients required dialysis for acute renal failure.

## Discussion

In Uttar Pradesh, the maternal mortality ratio is higher than the national maternal mortality ratio. So this study was conducted to find out the incidence of near-miss events based on the Government of India Health and Family Welfare Guidelines 2014 and to compare common causes responsible for maternal near-miss with maternal mortality at a rural tertiary care center that is in Uttar Pradesh, which can help in further improvement.

A study by Bansal et al. [7] in Bastar, Chhattisgarh, in 2016 showed a maternal near-miss incidence ratio of 11.9 /1000 live births, which is less than shown in our study. This may be because our center is in a rural area and covers most of the referral centers in these areas. Another study by Jain [8] in 2019 in Shivpuri, Madhya Pradesh, shows an MNM incidence ratio of 14.3, which was almost similar to that found in our study. Studies conducted in other countries, like in Nigeria by Akpan et al. [9], also showed a near-miss incidence ratio of 68.3/1000 live births and maternal near-miss ratio (MNMR):MMR of 1:8, which was higher than found in our study and showed better quality of health care services at their center.

In this study, the age group of 20 to 35 years was most commonly affected both by MNM and maternal mortality due to the fact that it is the most common reproductive age group so mostly complications occur in this age group. It is similar to the study by Bansal et al. [7] in Bastar, Chhattisgarh, in 2016, and Jain [8] in 2019 in Shivpuri, Madhya Pradesh.

Maternal mortality was significantly high (p-value 0.01) in unbooked women as compared to near-miss cases in this study. It may be because high-risk cases remain undiagnosed till complications develop and lead to mortality if not managed timely.

In this study, most of the near-miss cases were in the third trimester of pregnancy, post-partum and maternal mortality also mostly occurred in the same gestational age group, and near-miss cases were higher, which was statistically significant. It may be because most complications develop during the third trimester of pregnancy and our center is in a rural area and covers a large rural population so patients from all referral centers come to us. Sometimes, they deliver at other centers or at home and are then referred to our center or come on their own if any complication occurs. The study conducted at Ahmadabad by Mansuri et al. [10] and in Karnataka by Roopa et al. [11] also showed similarity in that most near-miss cases and mortality belonged to the third trimester of pregnancy.

The most common cause of a maternal near-miss was hypertensive disorder in this study, which was similar to the study done by Jain in 2019 [8] and the study by Mansuri et al. [10] in Ahmadabad, Gujrat, in 2018, whereas the study by Bansal et al. [7] in 2016 and Roopa et al. [11] in 2013 showed hemorrhage as the most common SAMM followed by hypertensive disorders in pregnancy. Another study done by Sultana et al. [12] in Karachi, Pakistan, showed hemorrhage, hypertensive disorder in pregnancy, and sepsis as the most common causes of near-miss events. Other studies done by Chikadaya et al. [13] in Zimbabwe and by Dessalegn et al. [14] in Ethiopia showed the most common cause of a near-miss as hemorrhage and hypertensive disorder in pregnancy followed by early pregnancy complications. In all these studies, hemorrhage and hypertensive disorder are the most important causes for both maternal morbidity and mortality, So to prevent mortality, we should work for early recognition and referral, if required, to improve outcomes.

## Conclusions

Hypertensive disorders in pregnancy and hemorrhage were the two leading causes of near-miss events followed by sepsis. Causes in near-miss and maternal mortality are almost similar. Our center provides good care. Maternal mortality mostly occurred in cases that reached delayed or moribund. It is necessary to boost referral services so that patients can reach the hospital in time and lives can be saved. Awareness-raising programs should be conducted for increasing the knowledge regarding antenatal check-ups needed, the need for blood investigation, and all the morbid conditions that can occur in pregnancy so that patients can contact the healthcare facility in time. As the near-miss analysis indicates the quality of health care and causes are almost similar to maternal mortality, its registry should be done.
